# Manipulating quantum information with spin torque

**DOI:** 10.1038/srep17912

**Published:** 2015-12-09

**Authors:** Brian Sutton, Supriyo Datta

**Affiliations:** 1 School of Electrical and Computer Engineering and Purdue Quantum Center, Purdue University, West Lafayette, IN, 47907.

## Abstract

The use of spin torque as a substitute for magnetic fields is now well established for classical operations like the switching of a nanomagnet. What we are describing here could be viewed as an application of spin torque like effects to quantum processes involving single qubit rotations as well as two qubit entanglement. A key ingredient of this scheme is the use of a large number of itinerant electrons whose cumulative effect is to produce the desired qubit operations on static spins. Each interaction involves entanglement and collapse of wavefunctions so that the operation is only approximately unitary. However, we show that the non-unitary component of the operations can be kept below tolerable limits with proper design. As a capstone example, we present the implementation of a complete CNOT gate using the proposed spin potential based architecture, and show that the fidelity under ideal conditions can be made acceptably close to one.

There has been enormous progress in the field of spintronics in the last twenty-five years driven by the discovery of diverse new phenomena that have made it possible to generate and detect useful levels of non-equilibrium spin currents and spin potentials even at room temperature. Some of these phenomena are finding applications in memory devices, see for example^1^, and a number of proposals have been put forth seeking to utilize them both for conventional logic and for neuromorphic logic^2^,^3^. Since spin is a primary entity envisioned for the physical realization of quantum bits, or qubits^4^,^5^, it seems natural to ask whether the modern advancements in generating non-equilibrium spin currents and voltages could be harnessed in building robust quantum computers.

One influential proposal for the design of a quantum computer^5^,^6^ is based on the use of donor and nuclear spins of phosphorous ^31^P atoms in a silicon matrix and much experimental progress has been reported in the last fifteen years towards the realization of structures that could enable proposals of this type^7^,^8^,^9^,^10^. Single qubits, 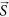, are selected and rotated using magnetic fields, while two qubit operations are realized by activating an effective exchange interaction 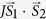 between them.

The use of spin torque as a substitute for magnetic fields is now well established for classical operations like the switching of a nanomagnet. Our primary objective in this paper is to show that “spin torque” like effects can be used to implement quantum processes involving single qubit initialization and rotation as well as two qubit entanglement. Qubit readout using ensemble-measurement can be implemented using the same architecture if a collection of identically initialized and transformed qubits, prepared using replicated physical structures, are available for measurement. Alternatively the proposed architecture could be used in conjunction with established single shot readout techniques^8^, especially for specific applications requiring multi-qubit state tomography or Bell state experiments.

In this paper we will first show that (1) all standard single qubit operations can be effected without any magnetic field through interactions of the form 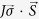 with the itinerant or “flying” non-equilibrium spin population 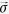 while (2) two qubit operations can be implemented through separate interactions of the form 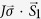 and 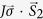 with the flying spin population 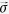. The latter process has been discussed earlier by several authors^11^,^12^,^13^,^14^,^15^,^16^,^17^,^18^,^19^,^20^,^21^ and we draw on this work, but there is a key distinction with the present work as explained in the next section.

## Materials and Methods

**Figure 1:**
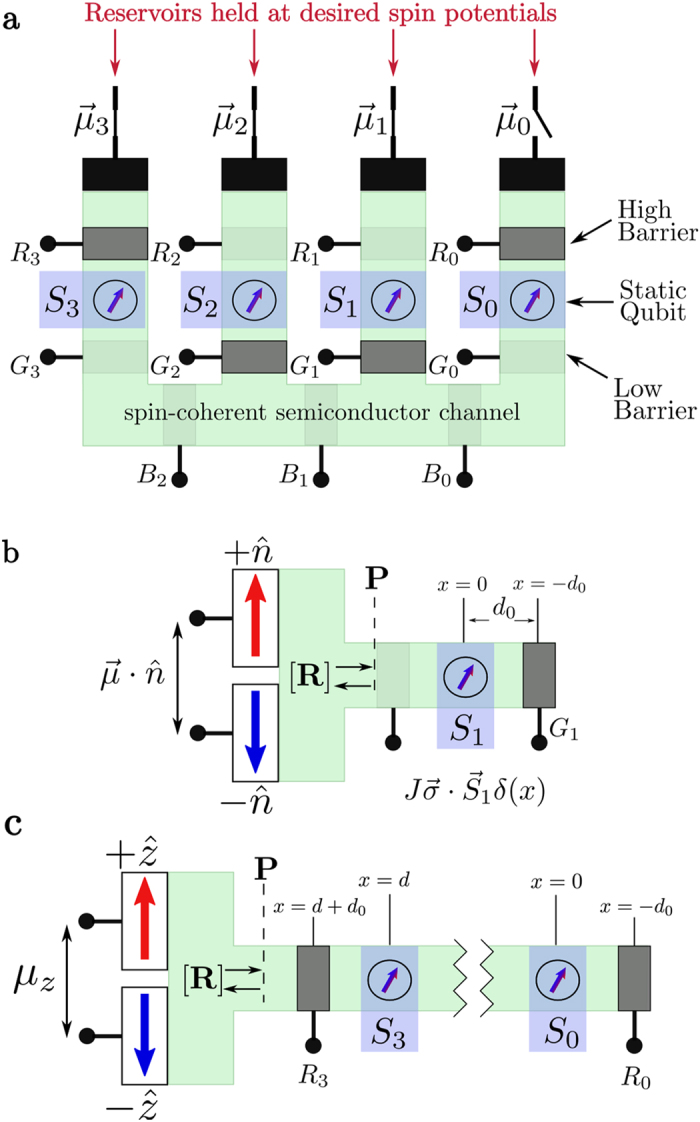
Quantum computing with “spin torque”. (a) Schematic showing the overall architecture with qubits *S*_1_ and *S*_2_ configured for single qubit operations and qubits *S*_0_ and *S*_3_ for two qubit operations. (**b**) Equivalent configuration for qubit *S*_1_ redrawn in one dimension. (**c**) Equivalent configuration for qubits 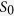 and 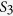 redrawn in one dimension.

The overall architecture we envision is shown schematically in Fig. 1(a) using four localized spins for illustrative purposes. A complete implementation could include additional qubits as well as multiple versions of the same qubit to allow ensemble readout. All qubits are embedded in a spin-coherent semiconductor channel so that the itinerant or “flying” (*f*) spins in the conduction band interact with the static 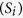 qubits located at 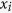 through an interaction of the form


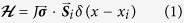


The static qubit could be a charge-neutral nuclear spin like ^29^Si with J representing the hyperfine interaction, or ^31^P where the donor level is used to mediate a hyperfine interaction, or it could be the electronic donor spin with J representing the exchange interaction, or perhaps a nanoscale magnet embedded in the semiconductor^22^,^23^.

Each qubit has gates, 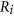 and 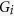, on either side that can be used to deplete the channel underneath to couple and decouple the qubits from the itinerant spins as desired for specific operations. The spin-coherent channel has additional gates, 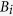, that provide isolation and direct the flow of electrons. These gates could be realized using top-gates^24^ or through contacts capable of modulating the electrostatics of the channel^10^. A general itinerant electron has a low probability of interacting with a static nuclear spin due to the minuscule size of the nucleus. In order to effect an interaction, the gates, 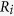 and 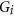, are used to produce standing waves for the itinerant electrons. These standing waves should in-turn provide the necessary wavefunction overlap to realize the coupled interaction with a static spin^25^.

The semiconducting channel could be realized using silicon^26^ or other material supporting coherent spin injection and transport^27^,^28^. Itinerant spins can be connected as desired to spin reservoirs held at specific spin potentials^29^ along the x, y or z directions. These can be generated using various well-established spintronic phenomena such as magnetic contacts^30^,^31^, the giant spin Hall effect^32^,^33^, or spin pumping^34^, at both low^35^ and room temperatures^36^,^37^. Integration of semiconductors with magnetic materials is a viable prospect^38^,^39^ and has been used in silicon double dot experiments to generate local magnetic fields^40^, establishing precedent for the prospect of device fabrication. Please see the discussion section for additional discussion of these topics along with a summary of the essential requirements for the proposal in Table 1.

Figure 1(a) shows qubits 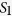 and 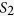 configured for single qubit operations and can be redrawn in one dimension as shown in Fig. 1(b). Qubits 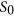 and 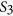 on the other hand are configured for two qubit operations and can be redrawn as shown in Fig. 1(c). Note that in either case the two reservoirs shown are purely conceptual; in practice these would likely be a single reservoir with a spin potential 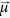 in some direction 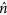. With a single contact under steady-state conditions there is no net current flow, however there is a continual exchange of electrons. Electrons are preferentially injected from the contact with spins in direction 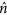 which interact with the static qubits and are then removed by the same contact. It is this flow of electrons to and from the same contact that drives the scattering phenomena described in the following sections.

The density matrix of the incident spins from this reservoir is given by 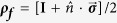, where **I** is the 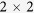 identity matrix and 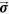 the Pauli spin matrices. The Kronecker product of this flying spin density matrix with the 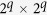 density matrix 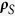 describing the q-qubit system (*q* = 1 for Fig. 1(b), *q* = 2 for Fig. 1(c)) gives the initial overall 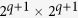 density matrix of the system. The initial density matrix gets modified to


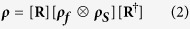


by the reflection process described by a 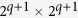 reflection matrix [**R**] which is computed taking into account the barrier(s) and the interaction of the itinerant spins with the qubits and depends on the specific structure at hand.

The reflected itinerant spins are returned to the spin reservoir 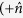 or 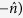 causing a collapse of the quantum state described by a partial trace of the density matrix over the flying spins represented by Trace_*f*_:


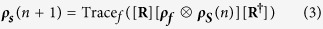


Equation (3) defines the basic approach we will use to model the quantum gates discussed in this paper. It provides a recursive relation expressing the q-qubit density matrix after interacting with 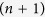 itinerant spins in terms of the density matrix after interacting with *n* itinerant spins. A time-independent model for quantum transport is used assuming that the time variation of signals is slow enough to be treated as quasi-static. For example, 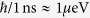 which is much smaller than other energy scales of interest.

The use of “flying spins” to manipulate static qubits has been discussed in the past^11^,^12^,^13^,^14^,^15^,^16^,^17^,^18^,^19^,^20^,^21^ and it has been noted that the reflection matrix [**R**] in equation (2) represents a unitary transformation suitable for quantum operations if the barriers at 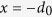 in Fig. 1(b,c) are large enough to reflect the incident electrons completely^20^,^21^, which we employ in this proposal. What is new about the present proposal’s method is the use of sequential interactions with a large number of itinerant electrons, each interaction involving a process of entanglement and reflection, equation (2), followed by a collapse, without post-selection, of the quantum state, equation (3), resulting in a deterministic, approximately unitary operation. Every interaction evolves the density matrix according to equation (3) which can be used iteratively to determine the final density matrix after interacting with a specified number of electrons.

Note that unlike equation (2), the collapse of the density matrix described by equation (3) is a non-unitary process and it may seem surprising that an overall operation involving a large number (*N*) of such non-unitary collapses could still be useful for implementing unitary transformations suitable for quantum computing. However, we will show that with proper choice of parameters the degree of non-unitarity can be made arbitrarily small at the expense of speed.

We seek to show that the non-unitarity that is inevitable with multiple collapses can be held to acceptably low levels so that useful quantum gates can be implemented. This is established first for single qubit operations and then for two qubit operations using the basic approach embodied in equations (2) and (3). Finally, as a capstone example, we present the implementation of a complete CNOT gate using the proposed architecture, and show that the fidelity under ideal conditions can be made acceptably close to one.

## Results

### Single Qubit Operations

Figure 1(b) shows the basic configuration for a single qubit operation: in the following discussion we will assume that 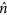 points along the z-direction, so that the reservoir injects electrons with + z spins and extracts both ±z spins. Every time an electron is injected it gets entangled with the static spin, while the extraction represents a measurement that collapses the quantum state of the static spin. We show in Supplementary Section A that after interaction with *N* electrons, the z-component of the static spin 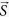 is given by


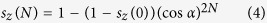


while the transverse component 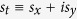 is given by


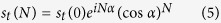


where *α* represents the effective interaction strength between the flying spin and the static qubit and is given by


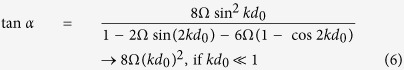


where *k* is the wavenumber of the itinerant electrons, *v* the corresponding velocity and


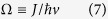


**Figure 2:**
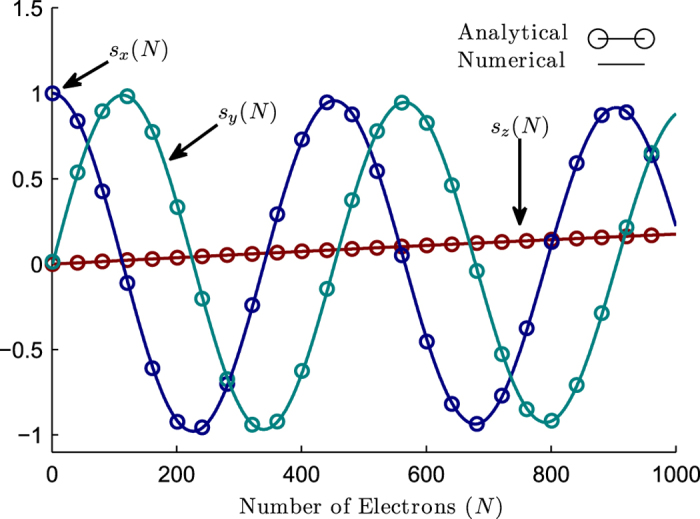
Single Qubit Rotation. Evolution of the spin of a single qubit initially pointing along x as it interacts with an increasing number of flying spins, *N*. The numerical results are described very well by the analytical solutions (4) and (5) described in the text.

Equations (4) and (5) describe our numerical results accurately, as evident from Fig. 2, and provide the basis for single qubit initialization and rotation respectively as described in the discussion section.

### Two Qubit Operations

Figure 1(c) shows the basic configuration for a two qubit operation which is very similar to that for a single qubit operation, Fig. 1(b), except that the channel has two embedded qubits instead of one. The overall approach is the same, based on equation (3), but the reflection matrix [**R**] is 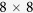 in size instead of 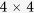, making the algebra less straightforward.

In the two qubit subspace we are seeking to implement a unitary transformation of the form


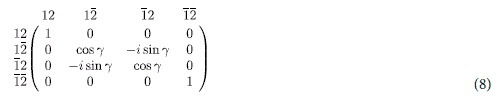


which could be viewed as a “rotation” in the 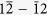 space: a rotation of 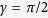 corresponds to a SWAP while 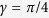 corresponds to the universal 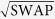 operation that we will use for the CNOT gate.

The approach used is based on the general principle of using itinerant spins as “messengers” that interact with the static spins through separate terms of the form 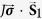, 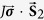. Each messenger causes a small rotation, and an overall rotation is achieved through the integrated effect of many messengers.

**Figure 3:**
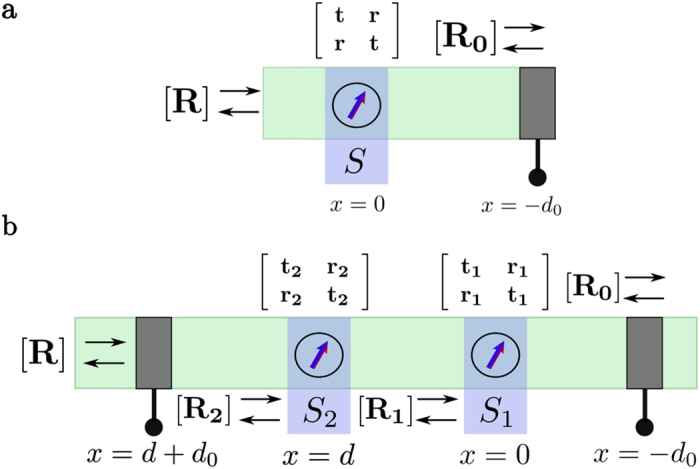
Single and Two Qubit Reflection Matrices. Reflection matrix [R] for (a) single qubit operations and (**b**) two qubit operations.

To see how this works we need the reflection matrix [**R**] which is calculated using an extension of the method used in Supplemental Section A. The interaction with each of the qubits is described by transmission and reflection matrices given by


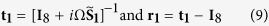



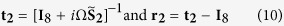


where 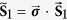 and 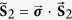 are 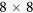 matrices (see Supplemental Section D for the explicit form of these matrices) describing the interactions of the itinerant spin with qubits 1 and 2 respectively. Note that the two qubit structure (Fig. 3(b)) has an additional barrier on the left with spin-independent transmission and reflection matrices given by


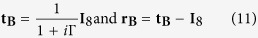


where 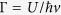 represents the barrier height normalized to 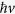, assuming a delta function barrier 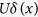.

The overall reflection matrix 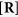 is calculated by repeated cascade of the reflection matrices for the structure in Fig. 3(b).


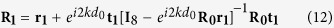



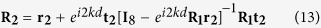



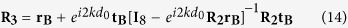


It is straightforward to use equations (12, 13, 14) to calculate the reflection matrix [**R**] for a given set of parameters Ω, Γ, *kd*, and *kd*_0_.

Regardless of the detailed choice of parameters, the overall reflection matrix is block-diagonal. Two of these are trivial 1 × 1 blocks, *f*12 and 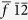, all upspin and all downspin, which remain unaffected by the interaction. The other two blocks are 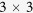 blocks involving 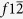, 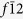, 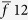 and 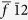, 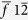, 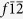 corresponding to (2 upspins + 1 downspin) and (1 upspin + 2 downspins) respectively. The overall block-diagonal reflection matrix [**R**] can be written in terms of five non-zero matrix elements *a, b, c*, 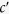 and 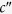 as shown below:


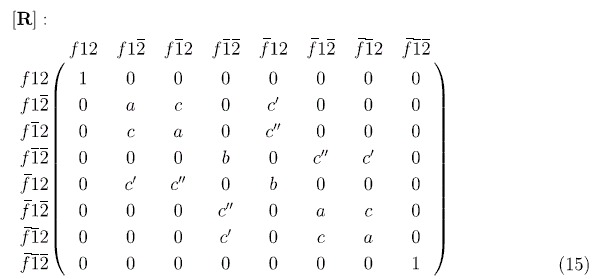


**Figure 4:**
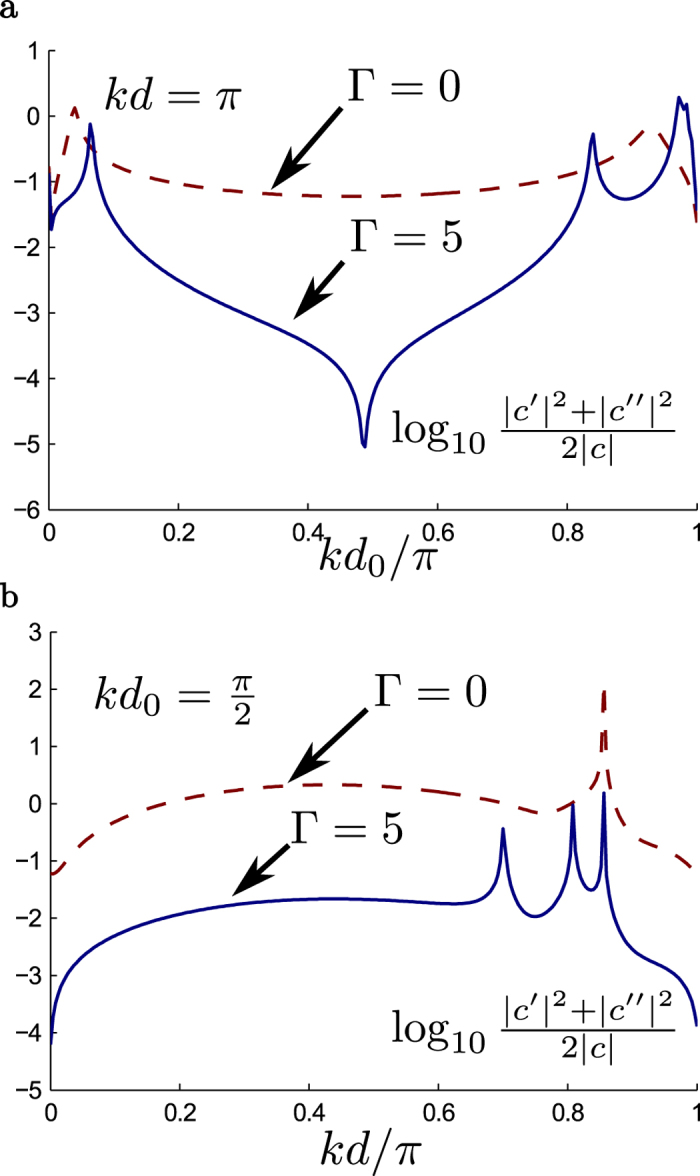
Figure of merit. Figure of merit for two qubit operations as a function of (a) *kd*_0_ for *kd = π* and (b) *kd* for *kd*_0_ = *π*/2 with Ω = 1 and Γ = 0 and Γ = 5.

Note that if 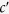, 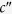 were zero, our reflection matrix would nearly provide the transformation we are looking for with term *c* providing the two qubit rotation *γ* in equation (8). But the terms 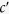 and 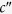 cause undesirable non-unitary effects leading to an average error probability


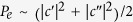


so that the error probability per unit rotation for the two qubit gate can be estimated from


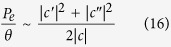


which is plotted in Fig. 4 as function of *kd* and 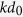 assuming 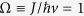, 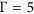. Note that with


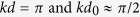


the error probability is quite small 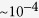.

To illustrate the importance of adding the additional semi-transparent barrier of strength Γ in front, we have also shown the results without it (Γ = 0) in dashed lines which show much larger error probability.

**Figure 5:**
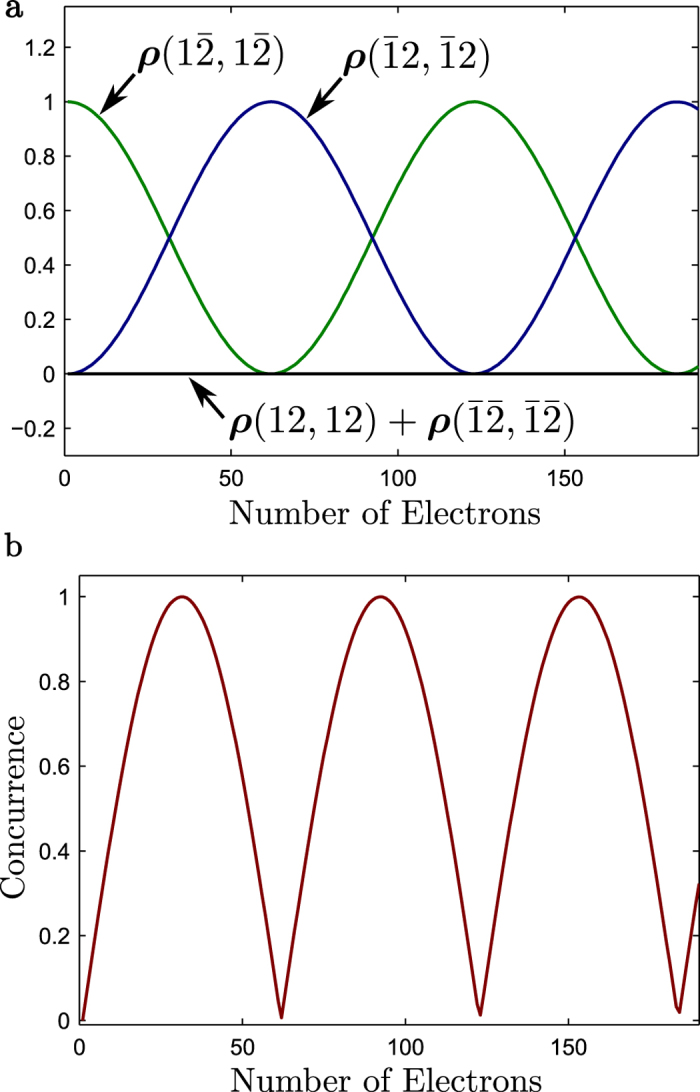
Two qubit rotation. (a) Two qubit rotation with *kd* = *π, kd*_0_ = *π*/2, Ω = 0.1, and Γ = 20. (**b**) Two qubit concurrence showing the oscillation of entanglement as a function of the number of incident electrons.

Figure 5(a) shows the evolution of the diagonal elements of the density matrix as it interacts with itinerant spins starting from an initial state with 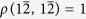. Note that entangled states with very high concurrence, see equation (10) from^41^, are obtained with the proper number of electrons (Fig. 5(b)).

### CNOT Implementation

**Figure 6:**
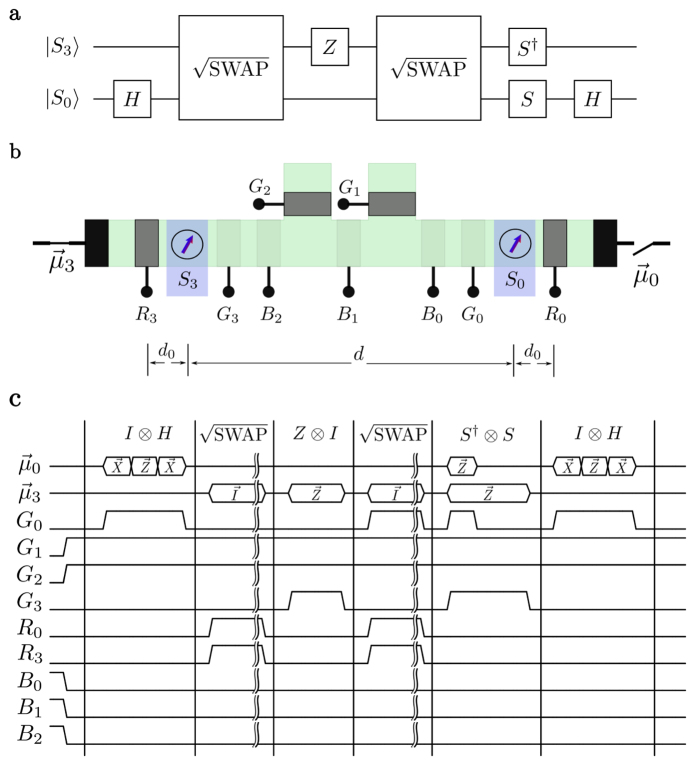
Controlled-NOT. (a) Circuit representation for CNOT based on the controlled-Z with Hadamard gates to obtain a controlled-X. (**b**) Physical picture for single and two qubit operations on 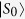 and 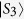 with electrostatically controlled gates. (**c**) Waveforms depicting the manipulations necessary for the various nets of Fig. (**b**) to perform a CNOT operation between 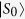 and 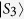.

We end this section with an example of another universal two qubit quantum gate, the CNOT, implemented using the spin potential-based architecture described here. Figure 6(a) shows a CNOT gate in terms of elementary single qubit and two qubit operations^4^. The circuit can be realized using the basic structure shown in Fig. 1(a) gated appropriately to obtain two qubits embedded in a spin coherent channel. These qubits can be selectively connected or disconnected from contacts held at specified spin potentials.

The timing diagram in Fig. 6(c) shows the sequence of single qubit and two qubit operations needed to implement the required gate:

1. H-gate: This involves 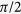 rotations of 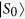 around the x, z, and x axes in sequence. The barrier gates 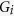, 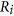 and 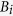 are used to isolate 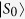 which is then rotated by connecting the 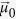 terminal to reservoirs with x and z-directed spin potentials, each for the length of time needed to provide the 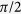 rotation.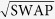 gate: This involves a two qubit rotation on 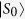 and 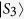 of the type discussed in the previous section. This gate is realized with the use of an unpolarized spin potential for the length of time appropriate for a 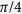 “rotation” (eq. (8)). Note the use of barrier gates *R*_0_ and *R*_3_ to implement the completely reflective barrier and the semitransparent barrier respectively.
3. Z-gate: This rotation of qubit 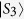 around the z-axis is achieved by using gate 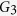 for isolation and then connecting to a z-directed spin potential for an appropriate duration.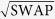 gate: same as step 2.
5. S and S^†^ gates: This involves a 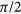 rotation of 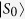 and a 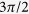 rotation of 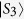, both around the z-axis.
6. H-gate: same as step 1.

The complete CNOT gate was simulated with single qubit and two qubit operations as described earlier with 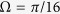 and 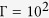. A 0.5% error in the desired values of *kd* and *kd*_0_ was assumed:


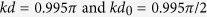


The fidelity of the gate was estimated using the prescription laid out by Trifunovic *et al*.^42^. The CNOT gate was simulated with each of the four (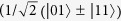 and 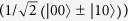 initial states that give rise to Bell states, and the fidelity of the final state 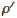 was evaluated by comparing to the ideal Bell state ***ρ***:


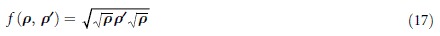


The minimum value of *f* was 99.8%.

## Discussion

Given the results of the previous section, we will assess the viability of the single and two qubit operations for fault tolerant quantum computing. After establishing the validity of these operations, we will then reflect on various aspects of the overall architecture.

### Single Qubit Operations

#### Single qubit initialization

Equation (4) tells us that a static spin can be initialized in a state with 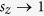, after interaction with a large number of flying spins 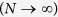. This is similar to the well-known Overhauser effect whereby nuclear spins get polarized through interaction with a spin reservoir driven out of equilibrium^25^,^43^ or other proposals for state purification using repeated measurements of a coupled quantum system with post-selection^44^,^45^,^46^.

#### Single qubit rotation

Equation (5) suggests the possibility of single qubit rotation by an angle 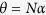 around the z-axis through interaction with the itinerant spins. Note, however, that in the process the spin is also attenuated by a factor 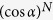 which is an undesirable side effect. For a given total rotation 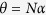 we can write the resulting error probability per unit rotation as


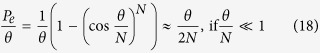


which shows that *P*_*e*_ can be made arbitrarily small for a given rotation *θ* by choosing a large *N* and hence a small *α*. The results in Fig. 2 were obtained with a relatively large value of *α* with 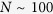 spins for a rotation of 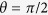 in order to make the non-unitary effects apparent with a large error probability. But with 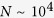, we have an error probability 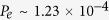.

The use of a large *N* also gives enhanced control over the process since each electron makes only a small difference to the result. A large N requires a small *α*, and equation (6) suggests a convenient mechanism for the control of *α*, namely by adjusting the effective distance *d*_0_ of the reflective barrier through the barrier voltage.

In this discussion, the polarization of the magnetic contact, 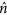, was taken as 100% in the z-direction. In practice, the polarization 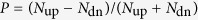 will be less than 100%. The angle of rotation will then be determined by 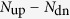, while the non-unitarity will depend on 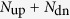, making the error probability in equation (18) larger by 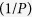. For example, a spin polarization of 0.01 would increase the error probability by 10^2^. In order to compensate for this increase in error, we would need 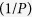 times more electrons, 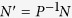, which could be accomplished by making *α* smaller. In silicon, spin polarizations an order of magnitude larger than 0.01 have been obtained at low temperatures^47^ with promising progress at room temperature^35^.

#### Single qubit readout

Note that the interaction we just discussed also provides a mechanism for readout if we have multiple replicas of each qubit available so that ensemble measurements can be made, similar to^17^. We could measure the average spin current *I*_*sz*_ that flows initially at the terminals


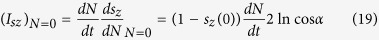


and deduce 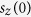 from it. Similarly the initial spin in the x- and y-directions can be obtained by measuring the spin current that flows when connected to a spin reservoir with an x- and y-component respectively and using equations (4, 5). Knowing 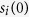, *i* = x, y, z, we can write down the initial density matrix as follows:


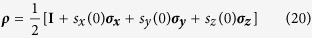


Ensemble measurement techniques provide a mechanism to obtain the expectation value of a given qubit and may be useful for certain computations^48^. However, as the architecture is compatible with single-shot readout methods, replicated physical structures are a non-essential aspect of the proposal.

### Two Qubit Operations

The direct approach to implementing a two qubit rotation is through an interaction of the form 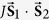 between the two static spins as, for example, in the Kane architecture^5^ or more recently in the quantum dot approaches proposed by Trifunovic et al.^42^,^49^. Using the proposed itinerant spin approach to mediate an effective exchange interaction leads to an inherent imperfection in the gate operations. These imperfections arise as a result of the undesirable coefficients 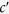 and 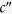 of (15) due to the interactions 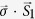, 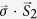. This is apparent if we compare the matrix representation of these operators with [**R**] in equation (15). On the other hand, the desirable coefficient *c* arises from product terms of the form


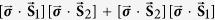


which are independent of the spin of the itinerant electrons, 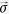, so that the two qubit operation, unlike the single qubit operations, does not require a spin potential; an ordinary unpolarized reservoir should be fine. The lack of phase symmetry of Eq. (15) for up and down flying spins, and hence imperfection in the 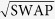, is somewhat mitigated with the use of un-polarized itinerant spins as, on average, the 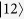 and 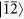 states will pick up the same overall phase.

The fidelity of the two-qubit 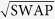 gate is strongly dependent on the height of the initial barrier, Γ. For 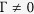, the enhancement in gate fidelity, being due to multiple reflection, is wavelength dependent, and hence *k* dependent. At low temperatures the relevant *k* is the Fermi wavevector *k*_*f*_ corresponding to the Fermi energy which is related to the electron density *n*_*s*_^50^:


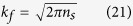


In general, however, a thermal average over wavevectors is involved and the degree of enhancement from multiple reflections will be averaged accordingly.

It should also be noted that since the reduced error probability with 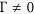 comes from multiple coherent reflections, this gain in performance can be expected to be more sensitive to processes that cause a loss of spin coherence. Such processes are ignored in our present model.

Finally, the fidelity of the complete CNOT implementation of 99.8% is sufficient for fault tolerant quantum computing^42^.

### Architecture

The proposed architecture has a number of features that may be advantageous for building a quantum computer in semiconductor based architectures. The itinerant spins generate localized magnetic fields for any target qubit, providing individual qubit selectivity, parallel operation, and qubit isolation. This localized field generation removes the need for AC electric and magnetic fields used for nuclear magnetic resonance (NMR) and electron spin resonance (ESR). Removing the need for these magnetic fields reduces the complexity of system design for qubit manipulation.

External DC magnetic fields can be eliminated if perfect half-metallic contacts can be obtained to produce 100% spin polarized currents suitable for high-fidelity qubit initialization. These ideal spin currents are currently difficult to realize experimentally, and an alternative initialization method is needed in the near-term. Alternatively, this architecture is compatible with existing approaches that leverage an external magnetic field to produce Zeeman splitting such that single qubit initialization and readout can be accomplished with single electron transistors.

Using itinerant spins for gate operations must be compatible with the decoherence times of the qubits. There have been significant recent advances in the long-term storage of quantum information in semiconductor systems based around donor and defect spins with experimental results of ^31^P nuclear spin T_2_ times ~30 seconds^24^, and high-purity silicon donor electrons with T_2_ times ~seconds^51^,^52^. These long decoherence times are much greater than the operation time of the quantum gates driven by itinerant spins: a current of 160 nA can deliver 10^4^ electrons, sufficient for a qubit rotation, in ~10 ns. Spin lifetimes of over 500 ns at 60 K have been reported in undoped Si^53^.

While single qubit gates in the architecture require the use of spin polarized currents and hence the integration of magnetic materials, two qubit gates can be realized with traditional contacts that produce un-polarized spin currents. Furthermore, these two qubit gates can be used to obtain non-local entanglement between selective qubits, a limitation of nearest neighbor proposals. The degree of non-locality will be limited by the spin coherent transport length of the itinerant quasi-particles which, depending on the material and the temperature, can range from tens of nanometers to tens of microns. However, coupling to channel contacts along with multiple reflections from barriers, increasing the effective transport length of the itinerant spins, will limit their range. As long as spin coherence can be maintained over a gate length (see Fig. 1) between two qubits, it should be possible to entangle them.

The use of a large number of itinerant spins to effect a given qubit operation allows fine tuning and control since a deviation of one electron represents a small error in a process involving, say, 10^4^ electrons. We envision controlling the actual number of electrons using gates to connect or disconnect the qubits from the itinerant spins as desired. The use of all-electrical control of qubits is beneficial for producing a scalable architecture using semiconductor based qubits. Other proposals for all-electrical control exist, however, these proposals are largely based on spin-orbit interactions and are likely to be susceptible to charge noise^54^.

Many proposals for qubit manipulations require sensitive gate control and the ability to manipulate single electrons. Here, the control for gate operations is based on a large number of electrons drawn from a reservoir which can be controlled accurately. Additionally, the architecture does not require a bound donor electron to perform nuclear spin manipulation which may provide a path for higher temperature operation^55^. As a further example of the reduction in control necessary to implement the architecture, the gate operations do not require precise placement of donors in the lattice.

Nevertheless, there are a number of challenges that are incurred by the architecture. Prominently is the inherent loss of gate fidelity as a result of repeated measurement. This loss of gate fidelity requires error correction even before other sources of decoherence and dephasing are considered. Magnetic material integration into semiconductor processing is another obstacle that must be overcome for experimental realization of high-fidelity initialization, single qubit gates, and ensemble qubit readout. There has been progress towards integration of these materials into fabrication processes as a result of modern magnetic memory technologies, however, this integration is still emerging and does not have mainstream adoption. Additionally, an interaction of the form given by (1) was assumed throughout the proposal. Based on this assumption, a more detailed exploration of the interaction between conduction-band flying electrons and donor-based nuclear spins is warranted.

In summary, we have outlined a quantum computing architecture based on the use of non-equilibrium spin potentials enabled by modern spintronics to perform all basic qubit operations including initialization, arbitrary single qubit rotation, single qubit readout, and two qubit rotation on selected pairs of qubits. A key feature of our architecture is the use of repeated entanglement with itinerant electrons and a subsequent collapse of the quantum state. The latter process is non-unitary, but we have shown that the overall non-unitary component can be kept below tolerable limits with proper design. Finally we presented the implementation of a complete CNOT gate using the proposed spin potential based architecture, and showed that the fidelity under ideal conditions is acceptable for fault tolerant quantum computing. This all-electrical architecture provides a means of qubit control for semiconductor donor systems without the use of magnetic fields while providing qubit selectivity and isolation, and non-local two qubit operation. Future research may include further investigation of the two qubit entanglement operation, numerical modeling of representative experimental structures, and investigation of the interaction between conduction band flying electrons and donor-based nuclear spins. Experimental investigation of the operations described herein would be valuable to assess the validity of the proposal.

## Additional Information

**How to cite this article**: Sutton, B. and Datta, S. Manipulating quantum information with spin torque. *Sci. Rep.* **5**, 17912; doi: 10.1038/srep17912 (2015).

## Supplementary Material

Supplementary Information

## Figures and Tables

**Table 1:**
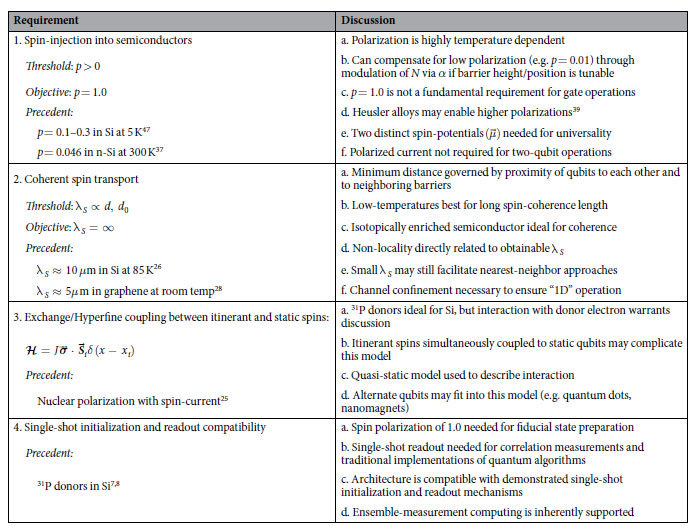
Essential Requirements.

There are a number of requirements that must be satisfied in order for the proposed architecture to be viable. Shown in the table are a few of the most essential requirements for the proposal along with precedent for their satisfaction.
