# Supplementary material for: Manipulating quantum information with spin torque
Source: Sci Rep. 2015 Dec 9;5:17912. doi: 10.1038/srep17912 (PMC4673422; doi:10.1038/srep17912)
Supplement: Supplementary Information [file srep17912-s1.pdf]

Manipulating quantum information with spin torque

Brian Sutton*[1, a)] and Supriyo Datta*[1, b)]
School of Electrical and Computer Engineering, Purdue University, West Lafayette, IN, 47907

(Dated: November 1, 2015)

[a)]bmsutton@purdue.edu
[b)]datta@purdue.edu

SUPPLEMENTARY INFORMATION

Appendix A: Derivations of equations (4) and (5)

Since the discussion of single qubit operations using non-equilibrium spins is all based on equations (4) and (5) let us now describe how these are obtained. Our starting point is equation (3) which relates the qubit density matrix after it has interacted with $n+1$ spins to the earlier density matrix after it had interacted with n spins.

The density matrix for the qubit can be written as

$$\boldsymbol{\rho_s}(n) = \frac{1}{2}\begin{bmatrix} 1+s_z(n) & s_t^*(n) \\ s_t(n) & 1-s_z(n) \end{bmatrix} \tag{A1}$$

while the itinerant z-directed spins are describe by a density matrix $\boldsymbol{\rho_f} = [\,1\;\;0\,]^{\mathrm{T}}[\,1\;\;0\,]$. We can write from equation (2)

$$\boldsymbol{\rho} = \frac{1}{2}\mathbf{R}\begin{bmatrix} 1+s_z(n) & s_t^*(n) & 0 & 0 \\ s_t(n) & 1-s_z(n) & 0 & 0 \\ 0 & 0 & 0 & 0 \\ 0 & 0 & 0 & 0 \end{bmatrix}\mathbf{R}^\dagger \tag{A2}$$

To find the reflection matrix [$\mathbf{R}$] we note that it is related to the reflection matrix [$\mathbf{R_0}$] for the barrier and the reflection and transmission matrices [$\mathbf{r}$], [$\mathbf{t}$] describing the interaction of the itinerant spins with the static qubit (Figure 3(a))

$$\mathbf{R} = \mathbf{r} + e^{i2kd_0}\mathbf{t}\left[\mathbf{I}_4 - e^{i2kd_0}\mathbf{R_0}\mathbf{r}\right]^{-1}\mathbf{R_0}\mathbf{t} \tag{A3}$$

Here $\mathbf{I}_4$ is the 4×4 identity matrix and the matrices [$\mathbf{r}$] and [$\mathbf{t}$] can be written as (see Supplementary Section B)

$$\mathbf{t} = \left[\mathbf{I}_4 + i\Omega\tilde{\mathbf{S}}\right]^{-1} \text{ and } \mathbf{r} = \mathbf{t} - \mathbf{I}_4 \tag{A4}$$

$\tilde{\mathbf{S}}$ being the 4×4 matrix, $\vec{\boldsymbol{\sigma}}_{\boldsymbol{f}}\cdot\tilde{\mathbf{S}}$, describing the basic itinerant spin(f)-qubit(s) interaction in equation (1):

$$\tilde{\mathbf{S}} = \begin{array}{c} \\ fs \\ f\bar{s} \\ \bar{f}s \\ \bar{f}\bar{s} \end{array}\!\!\begin{array}{c} \begin{array}{cccc} fs & f\bar{s} & \bar{f}s & \bar{f}\bar{s}) \end{array} \\ \begin{pmatrix} 1 & 0 & 0 & 0 \\ 0 & -1 & 2 & 0 \\ 0 & 2 & -1 & 0 \\ 0 & 0 & 0 & 1 \end{pmatrix} \end{array} \tag{A5}$$

Assuming the barrier reflection coefficient to be given by $\mathbf{R_0} = -[\mathbf{I}_4]$ and using equations (A4) and (A5), we obtain the desired reflection matrix [$\mathbf{R}$] from equation (A3) (see Supplementary Section C for details)

$$\mathbf{R} = \begin{array}{c} \\ fs \\ f\bar{s} \\ \bar{f}s \\ \bar{f}\bar{s} \end{array}\!\!\begin{array}{c} \begin{array}{cccc} fs & f\bar{s} & \bar{f}s & \bar{f}\bar{s} \end{array} \\ \begin{pmatrix} e^{-i\alpha} & 0 & 0 & 0 \\ 0 & \cos\alpha & -i\sin\alpha & 0 \\ 0 & -i\sin\alpha & \cos\alpha & 0 \\ 0 & 0 & 0 & e^{-i\alpha} \end{pmatrix} \end{array} \tag{A6}$$

where α is given by equation (6) as stated earlier.

Using $[\mathbf{R}]$ in equation (3) we have for $\boldsymbol{\rho}$ (dropping the argument n for clarity)

$$\frac{1}{2}\begin{bmatrix} 1+s_z & s_t^* \cos\alpha e^{-i\alpha} & iS_t^* \sin\alpha e^{-i\alpha} & 0 \\ s_t \cos\alpha e^{i\alpha} & (1-s_z)\cos^2\alpha & i(1-s_z)\cos\alpha\sin\alpha & 0 \\ -iS_t \sin\alpha e^{i\alpha} & -i(1-s_z)\cos\alpha\sin\alpha & (1-s_z)\sin^2\alpha & 0 \\ 0 & 0 & 0 & 0 \end{bmatrix}$$

which upon collapsing, as indicated in equation (3), gives the static qubit density matrix after interacting with $(n+1)$ spins

$$\boldsymbol{\rho_s}(n+1) = \frac{1}{2}\begin{bmatrix} (1+s_z(n)) + (1-s_z(n))\sin^2\alpha & s_t^*(n)\cos\alpha e^{-i\alpha} \\ s_t(n)\cos\alpha e^{i\alpha} & (1-s_z(n))\cos^2\alpha \end{bmatrix}$$

which by definition must equal

$$\boldsymbol{\rho_s}(n+1) = \frac{1}{2}\begin{bmatrix} 1+s_z(n+1) & s_t^*(n+1) \\ s_t(n+1) & 1-s_z(n+1) \end{bmatrix}$$

so that we can write

$$s_z(n+1) = s_z(n) + (1-s_z(n))\sin^2\alpha \tag{A7}$$

$$s_t(n+1) = s_t(n)\cos\alpha e^{i\alpha} \tag{A8}$$

It is straightforward to check that the solutions stated earlier in (4) and (5) satisfy the recurrence relations in equations (A7) and (A8).

Appendix B: Equation (A4)

$\tilde{\mathbf{S}}$ can be written in the standard basis as

$$\tilde{\mathbf{S}} = \vec{\boldsymbol{\sigma}}_{\boldsymbol{f}} \cdot \vec{\boldsymbol{S}} = \begin{bmatrix} 1 & 0 & 0 & 0 \\ 0 & -1 & 2 & 0 \\ 0 & 2 & -1 & 0 \\ 0 & 0 & 0 & 1 \end{bmatrix} \tag{B1}$$

Under basis transformation $\mathbf{U}$ to the $|fs\rangle$, $(|f\bar{s}\rangle + |\bar{f}s\rangle)/\sqrt{2}$, $(|f\bar{s}\rangle - |\bar{f}s\rangle)/\sqrt{2}$, $|\bar{f}\bar{s}\rangle$ basis, this matrix becomes diagonal.

$$\mathbf{U}^\dagger\tilde{\mathbf{S}}\mathbf{U} = \begin{array}{c|cccc} & fs & \frac{f\bar{s}+\bar{f}s}{\sqrt{2}} & \frac{f\bar{s}-\bar{f}s}{\sqrt{2}} & \bar{f}\bar{s}) \\ \hline fs & 1 & 0 & 0 & 0 \\ \frac{f\bar{s}+\bar{f}s}{\sqrt{2}} & 0 & 1 & 0 & 0 \\ \frac{f\bar{s}-\bar{f}s}{\sqrt{2}} & 0 & 0 & -3 & 0 \\ \bar{f}\bar{s} & 0 & 0 & 0 & 1 \end{array} \tag{B2}$$

As a result the scattering problem can be treated as four independent one-dimensional channels with transmission matrix $\mathbf{U}^\dagger\mathbf{t}\mathbf{U}$ given by

$$\mathbf{U}^\dagger\mathbf{t}\mathbf{U} = \begin{bmatrix} (1+i\Omega)^{-1} & 0 & 0 & 0 \\ 0 & (1+i\Omega)^{-1} & 0 & 0 \\ 0 & 0 & (1-3i\Omega)^{-1} & 0 \\ 0 & 0 & 0 & (1+i\Omega)^{-1} \end{bmatrix}$$

$$\mathbf{U}^\dagger\mathbf{t}\mathbf{U} = [\mathbf{I_4} + i\Omega(\mathbf{U}^\dagger\tilde{\mathbf{S}}\mathbf{U})]^{-1}$$

Which yields equation (A4)

$$\mathbf{t} = [\mathbf{I_4} + i\Omega\tilde{\mathbf{S}}]^{-1} \tag{B3}$$

Appendix C: From equations (A3), (A4), and (A5), to equations (A6) and (6)

We can write from equation (A3) with $\mathbf{R_0} = -\mathbf{I}_4$

$$\mathbf{R} = \mathbf{r} - e^{i2kd_0}\mathbf{t}\left[\mathbf{I}_4 + e^{i2kd_0}\mathbf{r}\right]^{-1}\mathbf{t} \tag{C1}$$

where

$$\mathbf{t} = \left[\mathbf{I}_4 + i\Omega\tilde{\mathbf{S}}\right]^{-1} \text{ and } \mathbf{r} = \mathbf{t} - \mathbf{I}_4 \tag{C2}$$

We can diagonalize $\mathbf{r}$ and $\mathbf{t}$ as well as $\mathbf{R}$ by diagonalizing $\tilde{\mathbf{S}}$:

$$\tilde{\mathbf{S}} = \vec{\boldsymbol{\sigma}} \cdot \vec{\mathbf{S}} = \begin{array}{c} \\ fs \\ \frac{f\bar{s}+\bar{f}s}{\sqrt{2}} \\ \frac{f\bar{s}-\bar{f}s}{\sqrt{2}} \\ \bar{f}\bar{s} \end{array} \begin{array}{c} \begin{array}{cccc} fs & \frac{f\bar{s}+\bar{f}s}{\sqrt{2}} & \frac{f\bar{s}-\bar{f}s}{\sqrt{2}} & \bar{f}\bar{s}) \end{array} \\ \begin{pmatrix} 1 & 0 & 0 & 0 \\ 0 & 1 & 0 & 0 \\ 0 & 0 & -3 & 0 \\ 0 & 0 & 0 & 1 \end{pmatrix} \end{array} \tag{C3}$$

so that

$$\mathbf{R} = \vec{\boldsymbol{\sigma}} \cdot \vec{\mathbf{S}} = \begin{array}{c} \\ fs \\ \frac{f\bar{s}+\bar{f}s}{\sqrt{2}} \\ \frac{f\bar{s}-\bar{f}s}{\sqrt{2}} \\ \bar{f}\bar{s} \end{array} \begin{array}{c} \begin{array}{cccc} fs & \frac{f\bar{s}+\bar{f}s}{\sqrt{2}} & \frac{f\bar{s}-\bar{f}s}{\sqrt{2}} & \bar{f}\bar{s}) \end{array} \\ \begin{pmatrix} R_T & 0 & 0 & 0 \\ 0 & R_T & 0 & 0 \\ 0 & 0 & R_S & 0 \\ 0 & 0 & 0 & R_T \end{pmatrix} \end{array} \tag{C4}$$

where

$$R_m = r_m - \frac{t_m^2 e^{2ikd_0}}{1 + r_m e^{2ikd_0}} \text{ , } m = T, S \tag{C5}$$

$$t_T = 1 + r_T = \frac{1}{1 + i\Omega} \tag{C6}$$

$$t_S = 1 + r_S = \frac{1}{1 - 3i\Omega} \tag{C7}$$

Using equations (C5), (C7), and (C6) we can write

$$R_m = -e^{2i\theta_m} \tag{C8}$$

where

$$\tan\theta_T = \frac{\sin kd_0}{\cos kd_0 + 2\Omega \sin kd_0} \tag{C9}$$

$$\tan\theta_S = \frac{\sin kd_0}{\cos kd_0 - 6\Omega \sin kd_0} \tag{C10}$$

Consider first the triplet coefficient R_T with $t_T = 1 + r_T$ and $r_T = (-i\Omega)/(1 + i\Omega)$ and let $P = e^{ikd_0}$. We can then write, dropping the sub-scripts for convenience

$$\begin{aligned}
R &= r - \frac{(1+r)^2 P^2}{1 + rP^2} \\
&= \frac{r + (r^2 - (1+r)^2)P^2}{1 + rP^2} = \frac{r - (2r+1)P^2}{1 + rP^2}\frac{1+i\Omega}{1+i\Omega} \\
&= \frac{-i\Omega - (1 - i\Omega)P^2}{1 + i\Omega - i\Omega P^2} = -\frac{i\Omega P^* + (1 - i\Omega)P}{(1+i\Omega)P^* - i\Omega P} \\
&= -\frac{Xe^{i\theta}}{Xe^{-i\theta}}
\end{aligned}$$

where

$$\begin{aligned}
Xe^{i\theta} &= i\Omega P^* + (1 - i\Omega)P \\
&= i\Omega(\cos kd_0 - i \sin kd_0) + (1 - i\Omega)(\cos kd_0 + i \sin kd_0) \\
&= (\cos kd_0 + 2\Omega \sin kd_0) + i \sin kd_0
\end{aligned}$$

which allows us to write

$$\tan \theta_T = \frac{\sin kd_0}{\cos kd_0 + 2\Omega \sin kd_0} \tag{C11}$$

Similarly, $\tan \theta_S$ can be found by replacing $\Omega \to -3\Omega$

$$\tan \theta_S = \frac{\sin kd_0}{\cos kd_0 - 6\Omega \sin kd_0} \tag{C12}$$

corresponding to equations (C9) and (C10) respectively. We can then write the reflection matrix as

$$\mathbf{R} = \begin{array}{c} \\ fs \\ \frac{f\bar{s}+\bar{f}s}{\sqrt{2}} \\ \frac{f\bar{s}-\bar{f}s}{\sqrt{2}} \\ \bar{f}\bar{s} \end{array} \begin{array}{c} \begin{array}{cccc} fs & \frac{f\bar{s}+\bar{f}s}{\sqrt{2}} & \frac{f\bar{s}-\bar{f}s}{\sqrt{2}} & \bar{f}\bar{s}) \end{array} \\ \begin{pmatrix} e^{-i\alpha} & 0 & 0 & 0 \\ 0 & e^{-i\alpha} & 0 & 0 \\ 0 & 0 & e^{+i\alpha} & 0 \\ 0 & 0 & 0 & e^{+i\alpha} \end{pmatrix} \end{array} \tag{C13}$$

where $\alpha \equiv \theta_S - \theta_T$ and we have dropped the overall phase factor of $e^{-i(\theta_T + \theta_S)}$. Transforming back to the original basis we have:

$$\mathbf{R} = \begin{array}{c} \\ fs \\ f\bar{s} \\ \bar{f}s \\ \bar{f}\bar{s} \end{array} \begin{array}{c} \begin{array}{cccc} fs & f\bar{s} & \bar{f}s & \bar{f}\bar{s} \end{array} \\ \begin{pmatrix} e^{-i\alpha} & 0 & 0 & 0 \\ 0 & \cos\alpha & -i\sin\alpha & 0 \\ 0 & -i\sin\alpha & \cos\alpha & 0 \\ 0 & 0 & 0 & e^{-i\alpha} \end{pmatrix} \end{array} \tag{C14}$$

Finally, making use of equations (C9) and (C10) we can write

$$\begin{aligned}
\tan\alpha = \tan(\theta_S - \theta_T) &= \frac{\tan\theta_S - \tan\theta_T}{1 + \tan\theta_S \tan\theta_T} \\
&= \frac{\frac{\sin kd_0}{\cos kd_0 - 6\Omega \sin kd_0} - \frac{\sin kd_0}{\cos kd_0 + 2\Omega \sin kd_0}}{1 + \frac{\sin^2 kd_0}{(\cos kd_0 - 6\Omega \sin kd_0)(\cos kd_0 + 2\Omega \sin kd_0)}} \\
&= \frac{(\cos kd_0 + 2\Omega \sin kd_0)\sin kd_0 - (\cos kd_0 - 6\Omega \sin kd_0)\sin kd_0}{\cos^2 kd_0 - 4\Omega \cos kd_0 \sin kd_0 - 12\Omega \sin^2 kd_0 + \sin^2 kd_0} \\
&= \frac{8\Omega \sin^2 kd_0}{1 - 4\Omega \cos kd_0 \sin kd_0 - 12\Omega \sin^2 kd_0}
\end{aligned}$$

which yields the result of (6)

$$\tan\alpha = \frac{8\Omega\sin^2 kd_0}{1 - 2\Omega\sin(2kd_0) - 6\Omega(1-\cos 2kd_0)} \tag{C15}$$

Appendix D: Matrices $\tilde{\mathbf{S}}_1$ and $\tilde{\mathbf{S}}_2$ in equations (9)(10)

$\tilde{\mathbf{S}}_\mathbf{1} = \vec{\boldsymbol{\sigma}} \cdot \vec{\mathbf{S}}_1 \rightarrow$

	$f12$	$f1\bar{2}$	$f\bar{1}2$	$f\bar{1}\bar{2}$	$\bar{f}12$	$\bar{f}1\bar{2}$	$\bar{f}\bar{1}2$	$\bar{f}\bar{1}\bar{2}$
$f12$	1	0	0	0	0	0	0	0
$f1\bar{2}$	0	1	0	0	0	0	0	0
$f\bar{1}2$	0	0	-1	0	2	0	0	0
$f\bar{1}\bar{2}$	0	0	0	-1	0	2	0	0
$\bar{f}12$	0	0	2	0	-1	0	0	0
$\bar{f}1\bar{2}$	0	0	0	2	0	-1	0	0
$\bar{f}\bar{1}2$	0	0	0	0	0	0	1	0
$\bar{f}\bar{1}\bar{2}$	0	0	0	0	0	0	0	1

$\tilde{\mathbf{S}}_\mathbf{2} = \vec{\boldsymbol{\sigma}} \cdot \vec{\mathbf{S}}_2 \rightarrow$

	$f12$	$f1\bar{2}$	$f\bar{1}2$	$f\bar{1}\bar{2}$	$\bar{f}12$	$\bar{f}1\bar{2}$	$\bar{f}\bar{1}2$	$\bar{f}\bar{1}\bar{2}$
$f12$	1	0	0	0	0	0	0	0
$f1\bar{2}$	0	-1	0	0	2	0	0	0
$f\bar{1}2$	0	0	1	0	0	0	0	0
$f\bar{1}\bar{2}$	0	0	0	-1	0	0	2	0
$\bar{f}12$	0	2	0	0	-1	0	0	0
$\bar{f}1\bar{2}$	0	0	0	0	0	1	0	0
$\bar{f}\bar{1}2$	0	0	0	2	0	0	-1	0
$\bar{f}\bar{1}\bar{2}$	0	0	0	0	0	0	0	1

$(\tilde{\mathbf{S}}_\mathbf{1}\tilde{\mathbf{S}}_\mathbf{2} + \tilde{\mathbf{S}}_\mathbf{2}\tilde{\mathbf{S}}_\mathbf{1})/2 \rightarrow$

	$f12$	$f1\bar{2}$	$f\bar{1}2$	$f\bar{1}\bar{2}$	$\bar{f}12$	$\bar{f}1\bar{2}$	$\bar{f}\bar{1}2$	$\bar{f}\bar{1}\bar{2}$
$f12$	1	0	0	0	0	0	0	0
$f1\bar{2}$	0	-1	2	0	0	0	0	0
$f\bar{1}2$	0	2	-1	0	0	0	0	0
$f\bar{1}\bar{2}$	0	0	0	1	0	0	0	0
$\bar{f}12$	0	0	0	0	1	0	0	0
$\bar{f}1\bar{2}$	0	0	0	0	0	-1	2	0
$\bar{f}\bar{1}2$	0	0	0	0	0	2	-1	0
$\bar{f}\bar{1}\bar{2}$	0	0	0	0	0	0	0	1
